# Outpatient treatment of the poisoned patients in Iran; may it be a feasible plan?

**DOI:** 10.1186/2008-2231-21-45

**Published:** 2013-06-05

**Authors:** Nasim Zamani, Omid Mehrpour

**Affiliations:** 1 Department of Clinical Toxicology, Loghman Hakim Hospital, Shahid Beheshti University of Medical Sciences, Tehran, Iran; 2 Atherosclerosis and Coronary Artery Research Center, Birjand University of Medical Science, Birjand, Iran; 3 Medical Toxicology and Drug Abuse Research Center (MTDRC), Birjand University of Medical Sciences (BUMS), Pasdaran Avenue, Birjand 9713643138, Iran

**Keywords:** Poisoning, Outpatient treatment, Poisoned patients

## Letter to the editor

Intentional and accidental poisoning or overdose is a serious concern in Iran. There are some reasons for this, first of which, is that Iran has the highest rate of opium addiction in the world. Also, there are no efficient rules and enough surveillance on selling the pharmaceutical drugs in the pharmacies in Iran and nearly all medications are available to the lay people as over the counter medications. So, there are so many poisoned patients admitted to the Iranian hospitals every year.

Treatment of the poisoned patients in Iran has been growing within the recent decades and there are some referral poison hospitals in each part of Iran. Although in many countries these patients refer to general hospitals and are managed by general or emergency medicine physicians under the supervision of the clinical toxicologists, in Iran the patients' falimies prefer to bring them to the tertiary poison hospitals and the physicians prefer to refer them to these centers because of the legal problems they generally encounter while managing these patients [1]. In Tehran, the capital city of Iran with a population of around 8,000,000, there are two referral poison hospitals: Loghman-Hakim poison hospital and Baharloo poison hospital. Loghman-Hakim Poison Hospital is the biggest tertiary poisoning hospital in Tehran and probably in the Iran and the middle east. This hospital gives the needed inpatient care to almost 100 poisoned patients a day. Other hospitals skilled in the management of the poisoned patients are Imam Reza poison hospital in Mashhad, Noor hospital in Isfahan, Razi hospital in Ahwaz, Farshchian hospital in Hamadan, and Ali-Asghar hospitals in Shiraz. Moreover, poisoning management centers in Birjand, Arak, Ghom, Ardabil, Tabriz, Sari, Bandar Abbas and Rasht are developing [2].

Every day, there are lots of requests from the extremely ill poisoned patients being in the long queue to be admitted to these centers from general hospitals. However, even with the 24-hour efforts of the medical and paramedical staff of these centers, it is not possible to respond to all of these requests mainly due to the limitations in the hospital beds. This is while the staff in emergency departments (EDs) of these centers generally have to admit the patients referring to them at the first step (including those who can out-patiently be managed) to their wards to overcome the overcrowdedness of their EDs.

On the other hand, the matter of outpatient treatment of the poisoned patients is a known entity worldwide that has been suggested to be safe and effective in many cases of poisoning even with ethanol, opioid, and carbon monoxide [3-13]. In special settings of these poisonings, a supervising toxicologist monitors the patients with mild to moderate intoxications for a median period of 3.8 hours until the patient is ready for discharge [14]. This may reduce the number of the patients who are admitted to the wards while they can be monitored for about 6–8 hours and then be discharged (for instance an asympthomatic TCA-poisoned patient). In the current setting, these patients are generally admitted to the wards because there is not enough space for them to be monitored in the emergency department. These patients have to be hospitalized and pay the cost of at least one night hospital stay without it being really necessary while taking the place of other critically ill patients. It can, therefore, be suggested that by allocating 10 or maybe 15 beds (even in the toxicology wards) to outpatient monitoring beds, the hospital can use the potential of admission of more poisoned patients in desperate need of advanced specialized and even critical care. Even, the psychiatry consultation routinely offered to the patients may be given in the same setting. This approach however is not applicable to the patients with baseline psychiatric disorders. This way, both the patients’ costs and emergency room overcrowding will be decreased while more critically ill patients can benefit from the care given by experienced medical staff with their specific talent to be managing poisoned patients.

## Competing interests

The authors declare that they have no competing interests.

## Authors’ contributions

NZ gave the idea and did bibliography and drafted the article. OM completed/edited/revised the article. Both authors read and approved the final manuscript.
